# Patients with adolescent idiopathic scoliosis perceive positive improvements regardless of change in the Cobb angle – Results from a randomized controlled trial comparing a 6-month Schroth intervention added to standard care and standard care alone. SOSORT 2018 Award winner

**DOI:** 10.1186/s12891-019-2695-9

**Published:** 2019-07-08

**Authors:** Sanja Schreiber, Eric C. Parent, Doug L. Hill, Douglas M. Hedden, Marc J. Moreau, Sarah C. Southon

**Affiliations:** 1 Faculty of Rehabilitation Medicine, University of Alberta, 3-48 Corbett Hall, 8205 114 Street, Edmonton, Alberta T6G2G4 Canada; 2 Department of Physical Therapy, University of Alberta, 2-50 Corbett Hall, Edmonton, Alberta T6G2G4 Canada; 3 University of Alberta, Alberta Health Services, Glenrose Rehabilitation Hospital, 10230 111 Ave NW, Edmonton, AB T5G 0B7 Canada; 4 Royal College of Physicians and Surgeons of Canada, 774 Echo Drive, Ottawa, ON K1S 5N8 Canada; 5 Department of Surgery, Faculty of Medicine & Dentistry, Stollery Children’s Hospital room 4D4.21, 8440 112 Street, Edmonton, AB T6G 2B7 Canada

**Keywords:** Physiotherapeutic scoliosis specific exercises, Schroth, Exercise, Cobb angle, Receiver operating characteristics curve, Minimal important difference (MID), Scoliosis, Spinal curvatures

## Abstract

**Background:**

The Cobb angle is proposed as the “disease process” outcome for scoliosis research because therapies aim to correct or stop curve progression. While the Scoliosis Research Society recommends the Cobb angle as the primary outcome, the Society on Scoliosis Orthopaedic and Rehabilitation Treatment prioritises, as a general goal, patient related outcomes over Cobb angle progression.

**Objective:**

To determine the threshold of change in the Cobb angle in adolescents with idiopathic scoliosis (AIS) who perceive improvement in a 6-months randomized controlled trial comparing a Schroth exercise intervention added to the standard of care to the standard of care alone.

**Methods:**

This is a secondary analysis of data from a randomized controlled trial of 50 patients with AIS, with curves ranging from 10° to 45°, with or without a brace. Participants with diagnoses other than AIS, surgical candidates or patients who had scoliosis surgery were excluded. The 6-month interventions consisted of Schroth exercises added to standard-of-care (observation or bracing) with daily home exercises and weekly therapy sessions (Schroth) or standard-of-care alone (Control).

The anchor method for estimating the minimal important difference (MID) in the largest Cobb angles (LC) was used. Patient-reported change in back status over the 6-month treatment period was measured using the Global Rating of Change (GRC) scale as anchor varying from − 7 (“great deal worse”) to + 7 (“great deal better”). Participants were divided into two groups based on GRC scores: Improved (GRC ≥2) or Stable/Not Improved (GRC ≤1). MID was defined as the change in the LC that most accurately predicted the GRC classification as per the receiver operating characteristic curve (ROC).

**Results:**

The average age was 13.4 ± 1.6 years and the average LC was 28.5 ± 8.8 °s. The average GRC in the control group was − 0.1 ± 1.6, compared to + 4.4 ± 2.2 in the Schroth group. The correlation between LC and GRC was adequate (r = − 0.34, *p* < 0.05). The MID for the LC was 1.0 °. The area under the ROC was 0.69 (0.52–0.86), suggesting a 70% chance to properly classify a patient as perceiving No Improvement/Stable or Improvement based on the change in the LC.

**Conclusion:**

Patients undergoing Schroth treatment perceived improved status of their backs even if the Cobb angle did not improve beyond the conventionally accepted threshold of 5°. Standard of care aims to slow/stop progression while Schroth exercises aim to improve postural balance, signs and symptoms of scoliosis. Given the very small MID, perceived improvement in back status is likely due to something other than the Cobb angle. This study warrants investigating alternatives to the Cobb angle that might be more relevant to patients.

**Trial registration:**

ClinicalTrials.gov, NCT01610908. Retrospectively registered on April 2, 2012 (first posted on June 4, 2012 - https://clinicaltrials.gov/ct2/keydates/NCT01610908)

## Background

The Cobb angle is the most frequently used “disease process” outcome used to monitor the status of adolescent idiopathic scoliosis (AIS) because the primary goal of treatment is to stop progression or correct the curves thereby preventing or attenuating possible health effects in adulthood [1]. The Scoliosis Research Society (SRS) recommendations standardizing the reports of non-operative research identifies the Cobb angle as the primary outcome [2]. The Society on Scoliosis Orthopaedic and Rehabilitation Treatment (SOSORT) consensus also recognizes the importance of monitoring the Cobb angle change in the conservative treatment. However, among treatment priorities, SOSORT ranks it behind aesthetics, quality of life, disability, back pain, psychosocial well-being, progression in adulthood and pulmonary function [3, 4].

The success of conservative treatments is most commonly defined by a curve progression of 5° or less in the Cobb angle at the end of the treatment [2]. This threshold recommendation between two radiographs comes from studies investigating the reliability of Cobb angle measurements where 5° was the standard error of measurement (SEM) reported for manual Cobb angle measurements and often interpreted as the smallest difference that could be reliably attibuted to true change rather than measurement error [5]. However, semi-automatic methods produce smaller SEM than the manual method [6]. In their systematic review of literature, Langensiepen and colleagues looked at the reproducibility of the new techniques of Cobb angle measurement, including, semi-automatic, automatic and using smart phone device, and found that the SEM for intra-rater reliability ranged from 0.74° to 3.4°, and for inter-rater reliability ranged from 1.2° to 5.1° [6]. Despite the common use of the Cobb angle, there is no conclusive evidence to suggest that the improved radiological outcomes also relate to other long-term improvements in those with scoliosis, such as function, quality of life, self-image and pain [7]. Moreover, Cobb angle is poorly related to the overal quality of life [8]. According to the Cochrane Collaboration, patient-related outcomes are important to collect and monitor in research in order to represent what is most important to patients about a condition and its treatment, and therefore should be studied in addition to disease process measurements [9].

Schroth physiotherapeutic scoliosis specific exercises (PSSE) consists of sensorimotor, postural and corrective breathing exercises individualized to the patient’s specific scoliosis curve pattern leading to correction of asymmetric posture in daily activities [10]. The main goals of the Schroth exercises is the recalibration of normal postural alignment through static/dynamic postural control to achieve the overall corrected postural stabilization [10]. The key component of the Schroth method is auto-correction, defined as the patient’s ability to reduce spinal deformity through active postural realignment of the spine in three dimensions [11]. Auto-correction is achieved through self-elongation and specific segmental corrections adapted to each curve pattern. SOSORT considers auto-correction to be the most important element of a scoliosis-specific exercise treatment [11]. Supervised Schroth exercises have been shown, in short term studies, to improve curve severity [12], pain [13], muscle endurance [12–15], self-image [13] and surface topography characteristics [16].

Patients undergo many changes during the course of AIS treatment. Some are small and some are large, but not all are valued or even perceived by patients. Many definitions have been proposed for clinically meaningful changes in the context of an intervention. Clinically significant effect has been defined as “the extent to which therapy moves someone outside the range of the dysfunctional population or within the range of the functional population.” [17]. Other suggest that clinically important changes refer to changes in a patient’s functioning that are meaningful for the individuals receiving a medical intervention [18]. The minimal important difference (MID) “denotes the smallest score or change in score that would likely be important from the patient’s or clinician’s perspective” [19].

When it comes to research on scoliosis, little is known about what changes patients perceive as important, including their perceptions and interpretations of changes in the Cobb angle.

Therefore, in a sample of patients undergoing 6-months of conservative treatment for AIS, we sought to determine the minimal change in the Cobb angle associated with perceived improvement in back status. As our sample consisted of participants receiving standard of care (observation and bracing) or standard of care plus exercises, our secondary objective was to determine if there was difference in the perceived change reported in each of the two groups.

## Methods

This is a secondary analysis of a previously published RCT [12]. The anchor method was used to determine the MID in the largest Cobb angle in response to 6 months of conservative treatment [20]. The anchor is an external indicator of change used to interpret the observed change in a target outcome [21]. In the present study, the global rating of change was used as anchor and the change in the largest Cobb angles was used as target outcome. To undergo MID estimation, the target and the anchor must be appreciably correlated for accurate prediction [19]. This is because the change in the target must reflect the change in the anchor [22].

### Participants

This study used data collected during a randomized controlled trial (RCT) designed to estimate the effect of a 6-month Schroth PSSE intervention added to standard of care compared to standard of care alone (observation or bracing) on curve severity (measured by Cobb angle) and quality of life (QOL) in patients with AIS. Details of trial and the primary outcomes have been published [20].

Data from the first 50 participants with AIS consecutively enrolled from the Stollery Children Hospital’s specialized scoliosis clinic were included in this study. Patients with AIS, between the ages of 10 and 18 years with curves measuring 10° to 45°, and who were able to attend weekly therapy visits were eligible. Those who were planning or had undergone surgery or had previously been treated with a brace were excluded. There were no exclusions related to skeletal maturity level. The study was approved by the University of Alberta Health Research Ethics Board (Pro00011552).

Spine radiographs were taken at the time of consent and randomization and study procedures began within 1 month afterwards.

### Interventions

The Schroth group received supervised Schroth PSSE intervention (designed and supervised by a certified Schroth therapist) consisting of five 1-h long individual sessions over the first 2 weeks, during which the participants were instructed in their home exercise program. This was followed by weekly 1-h visits and combined with their daily home exercises (30–45-min). The Schroth group participants were scheduled for a total of 27 lab visits in addition to standard of care (observation or bracing) as prescribed by a primary healthcare provider. A detailed description of the exercise prescription has been reported previously [12, 13, 20]. After the 6-month period, the participants were recommended to continue with the Schroth treatment, but were not supervised beyond the trial period.

The Control group participants received only the standard of care, consisting of observation or rigid bracing if the SRS bracing criteria [23] were met and the adolescent accepted that form of treatment. Bracing criteria are related to the magnitude of the Cobb angle, whether curve showed recent progression and level of skeletal maturity [23].

### Measurements

Cobb angles of the largest curves (LC) were measured on a coronal plane radiograph at baseline and at the 6-month follow-up between the most tilted upper and lower end vertebrae with a semi-automated digital measurement system. This system has demonstrated excellent reliability [24] with error (SEM) at ≤2.5° for the Cobb angle [24]. The intra-class correlation coefficient (ICC) for intra-rater reliability was 0.99 (95% Confidence interval 0.987–0.992) and 0.98 (CI 0.977–0.983) for the inter-rater reliability [24]. The largest Cobb angle (LC) was determined by an assessor blinded to the treatment group and timing of the radiograph. The change in the LC was calculated by subtracting the LC at baseline from the LC at 6 months; positive change indicates improvement in the Cobb angle, negative change indicates progression. The Cobb angle was used as the target measure in the analysis.

The Global Rating of Change (GRC) [22, 25] is a 15-point global rating scale estimating a person’s perceived change over time that ranges from − 7 (“A Very Great Deal Worse”) to + 7 (“A Very Great Deal Better”). On the GRC scale − 1 corresponds to “A tiny bit worse (almost the same)”, 0 to “About the same” and + 1 to “A tiny bit better (almost the same)” – these three scores are frequently combined and characterized as “no change” [26].

The GRC has been shown to have high test-retest reliability (ICC = 0.90, 27], and face validity (Pearson’s r = 0.72–0.90) when participants rated importance of change on a 15-point scale [27]. Evidence for its construct validity in children, comes from a 5-point GRC scale showing significant correlations with changes on the Child Perceptions Questionnaire (CPQ) [28]. These authors measured change in a sample of children aged 11 to 14 [28]. A 15-point GRC scale showed significant correlations with the Paediatric Asthma Quality of Life Questionnaire (PAQLQ) in a sample of children aged 11 to 17 [29]. These results suggest that asking children global questions about their symptoms elicits “valid and important information about their experience” [29].

Participants were not aware of their radiographic measurements when they completed the GRC. Further, to minimize the response bias that could have resulted from attention of the therapists in the Schroth group, the GRC was collected on a separate visit when no treatment was applied, or before the therapy session. Participants were presented with the following prompt customized to the research question at the 6-month evaluation: “*Please rate the overall condition of your back from the time you began the treatment until now”*. Two subgroups were created based on categorization of the raw GRC scale scores: Improved (+ 2 to + 7) and a combination of those Deteriorated (− 2 to − 7) or experiencing No Change (− 1, 0, + 1). To our knowledge, the measurement properties of collapsing the 15-point GRC scale to define two groups as we did, has not yet been reported in the literature.

### Statistical analysis

Descriptive statistics were calculated (mean and standard deviation, percentage) for the age at baseline, LC at baseline, LC at follow-up, and the change in LC for the whole sample, original trial groups and for the GRC subgroups (Table 1).

**Table 1:** Baseline characteristics of the study participants overall and by treatment groups

	Overall sample *N* = 50	Schroth exercises + Standard of care, *n* = 25	Standard of care, *n* = 25
Age (years, 95% CI)	13.0 (12.5–13.5)	13.5 (12.7–14.2)	13.3 (12.7–13.9)
Girls n (%)	47 (94)	23 (92)	24 (96)
Braced participants n (%)	34 (68)	17 (68)	17 (68)
Height (m, 95% CI)	1.60 (1.6–1.6)	1.60 (1.6–1.6)	1.60 (1.6–1.6)
Weight (kg, 95% CI)	48.2 (45.9–50.5)	45.9 (42.6–49.1)	50.5 (47.1–54.0)
Largest curve (°,95% CI)	28.5 (8.77)	29.1 (25.4–32.8)	27.9 (24.3–31.5)

*Abbreviations*: *n* number of participants, *CI* confidence interval

Authors recommend that the anchor should correlate at minimum (r ≥ 0.3) with the change score observed in the target outcome [22, 26, 30, 31].

First, the MID was calculated as the mean change in the LC for the participants who perceived their change on the GRC as important (“a little bit better” to “a great deal better” (+ 2 to + 7)).

Second, since the MID can also be defined as the change in LC which best distinguishes participants who considered themselves improved vs. deteriorated/stable, it was estimated using a receiver operating characteristics (ROC) curve. The ROC is a plot of the overall accuracy (sensitivity and specificity) of the predicted perceived change classification for each observed change in LC. First, the sensitivity and specificity to detect patients perceiving an improvement according to our GRC definition was calculated for every Cobb angle difference measured over the follow-up, and then sensitivity values were plotted against 1-specificity values for each Cobb angle change observed in our sample.

The amount of change in the LC with the best balance between sensitivity and specificity was identified as the MID cutoff because that is the point that best discriminates between improved and deteriorated/stable subjects (the curve coordinates closest to the top left corner of the ROC curve) [32, 33]. Specifically, ROC curves plot the sensitivity (true positive rate = $\frac{True\ Positive}{True\ Positive+ False\ Negative}$) against one minus the specificity (false positive rate = $\frac{False\ Positive}{False\ Positive+ True\ Negative}$) for each possible change in the LC outcome. The area under the ROC curve (AUC) was calculated to determine the ability of the LC to discriminate between improved and unchanged/deteriorated participants [32, 33]. An AUC of 0.50 indicates discrimination at the level of chance, while larger values indicate a better predictive ability of a model to properly discriminate between improved and not improved participants [32, 33]. All analyses were conducted using the statistical package IBM SPSS Statistics for Windows, Version 22.0. Armonk, NY: IBM Corp.

## Results

Of the 50 subjects enrolled in the trial, six did not complete the study and radiographs were missing for four, resulting in a total sample size of 40. The mean age and LC at baseline were 13.4 ± 1.6 years and 28.5 ± 8.8 °s, respectively for the overall sample. Age, sex, number of braced participants, height and weight were similar between the two trial groups but those in the Schroth group presented slightly larger LC at baseline [29.1 (25.4–32.8) vs 27.9 (24.3–31.5), respectively] (Table 1).

### MID estimation

#### MID estimated as a mean difference in LC in those with important perceived change

The correlation between change in the LC Curve and the GRC met the acceptable threshold for MID estimation (r = − 0.34, *p* < 0.05) [19]. In the Control group, the GRC at the 6-month follow-up was − 0.09 ± 1.59, compared to + 4.43 ± 2.2 in the Schroth group. All 12 participants presenting an improvement in their Cobb angle larger than the MID of 1^o^ and reporting an important perceived change (GRC ≥ 2) were in the Schroth group and their mean GRC at the 6-month follow-up was + 5.00 ± 1.51. (Table 2). There was only one participant in the Schroth group who did not perceive an important improvement. The mean GRC of the 15 Control participants who reported no important perceived improvement and did not show a MID change in the Cobb angle was 0.00 ± 0.70.

**Table 2:** Numbers of participants who improved or not their LC by more than 1° and reporting or not perceived improvement of GRC ≥ + 2 overall and in each therapy group

	Overall *N* = 40 (%)	Predictive values of the test, % (95% CI)	Schroth exercises + Standard of care, *n* = 20	Standard of care, *n* = 20
True positive: Improved based on GRC ≥ 2 and LC change > MID of 1^0^ (n, %)	12 (30%)	67 (41–86)	12 (60%)	0 (0%)
True negative: Not improved based on GRC < 2 and LC change ≤ MID of 1^0^ (n, %)	16 (40%)	73 (49–88)	1 (5%)	15 (75%)
False positive: Improved based on GRC ≥ 2 but LC change ≤ MID of 1^0^ (n, %)	6 (15%)	33 (14–59)	5 (25%)	1 (5%)
False negative: Not improved based on GRC < 2 but LC change > MID of 1^0^ (n, %)	6 (15%)	27 (12–50)	2 (10%)	4 (20%)

*GRC* Global Rating of Change, *LC* Largest Curve, *MID* Minimum Important Difference, *n* number of participants, *95% CI* 95% confidence interval

The resulting MID for those with GRC ≥ + 2 was a decrease of LC by 1.3 ° ± 3.98°. The mean change for those with GRC ≤1 was an increase of LC by 1.56° ± 4.09. There were eight (60%) participants in the Schroth and one (5%) in the Control group who improved by a value larger than our SEM of 2.5°. There were two (10%) in the Schroth and nine in the Control group (45%) who deteriorated by more than our SEM threshold of 2.5° (Table 3).

**Table 3:** Change in LC, GRC and presentation of improved and deteriorated participants beyond SEM

Overall *n* = 40	Schroth exercises + Standard of care; *n* = 20	Standard of care; *n* = 20
Change in largest curve overall (°) at 6-months (LC at 6 months – LC at baseline)	− 1.82 ± 3.21	2.33 ± 4.20
GRC (n ± SD) at 6-months	+ 4.43 ± 2.2	−0.09 ± 1.59
Change in largest curve with GRC ≥ + 2 (°)	−1.33 ± 3.98	NA
Change in largest curve with GRC ≤ + 1 (°)	NA	1.56 ± 4.09
Improved beyond SEM = 2.5 (n, %)	8 (40%)	0 (0%)
Deteriorated beyond SEM = 2.5° (n, %)	2 (10%)	9 (45%)

*Abbreviations*: *LC* Largest Curve, *GRC* Global Rating of Change, *TI* truly improved, *TD* truly deteriorated, *FI* falsely improved, *FD* falsely deteriorated, *n* number of participants with follow-up data, *SEM* standard error of measurement

#### MID estimated as the change in LC with best ability to detect participants perceiving improvement using ROC analysis

The AUC was 0.69 (95%CI 0.52–0.86), suggesting that a randomly chosen participant with AIS has a 69% chance to be properly classified as improved or not, according to their GRC score.

**Fig. 1:**
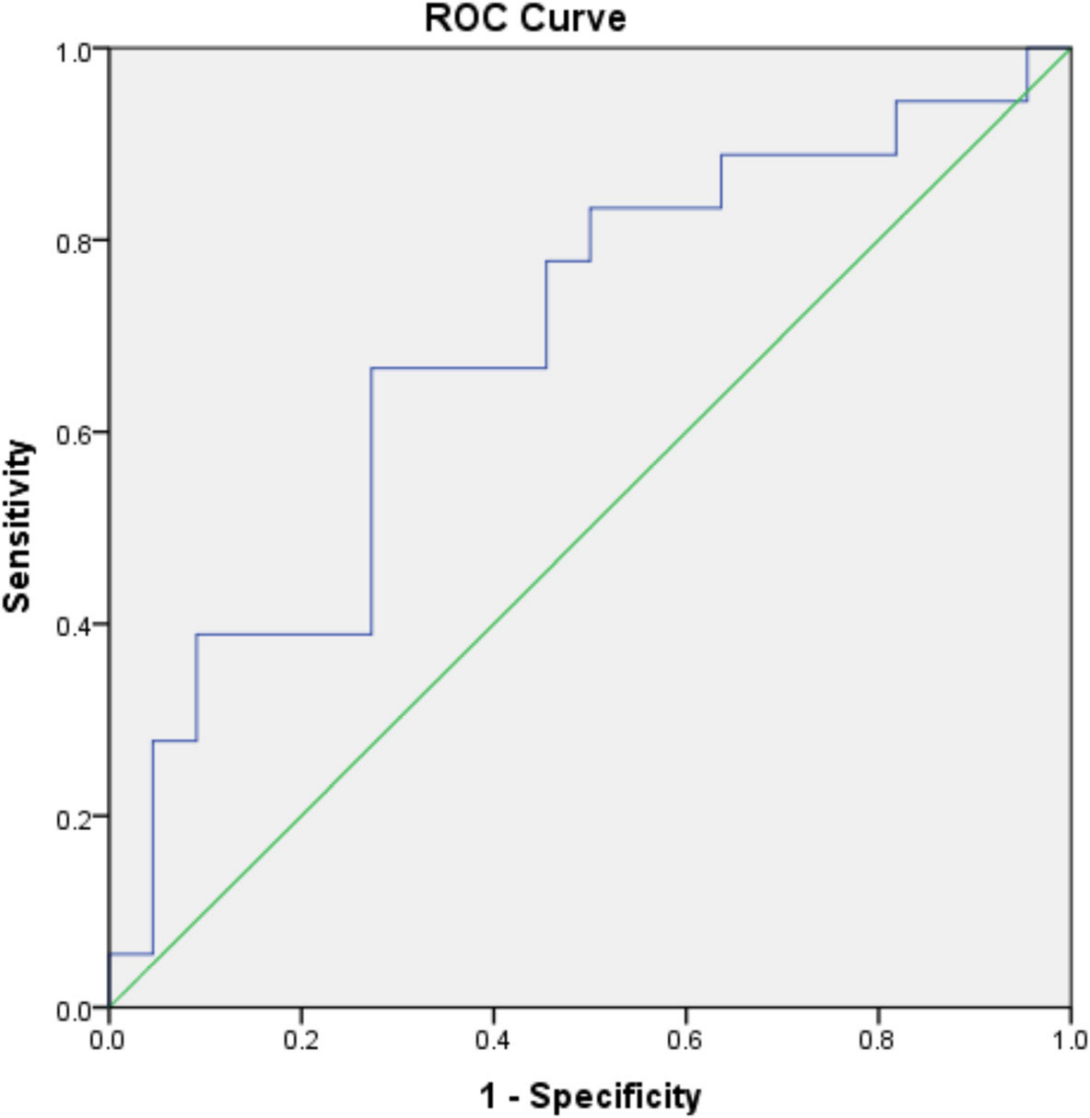
Receiver Operating Characteristics (ROC) Curve representing the balance between sensitivity (true positive rate) and one minus the specificity (false positive rate) for patients reporting perceived improvements (GRC ≥2) given various cut off points for change in the largest curve

The change in LC most accurately predicting perceived improvement was a 1.0° improvement (Fig. 1). This result suggests that participants truly observed a positive change when the LC improved by as little as 1°. Using this cut-off point, the sensitivity was 60%, the specificity was 75%, and overall 69% of the subjects were accurately classified as perceiving improvement or not.

There were overall 12 (30%) true positives presenting a change in LC over 1° and reporting perceived improvement of GRC ≥ + 2, of which all in the Schroth group. There were also, 16 (40%) overall true negatives (one in the Schroth and 15 in the Control group), with deteriorated LC reporting perceived deterioration or unchanged status (GRC ≤ + 1). There were overall six (15%) false positive whose LC improved but who did not perceive important improvement. There were also overall six (15%) false negative cases, perceiving an improvement but not showing an improvement in their LC. (Table 2).

## Discussion

Our study aim was to determine the change in Cobb angle required for AIS patients undergoing a 6-month Schroth RCT program to observe a positive change in their backs. In our sample 60% of the participants perceived improvement in their backs, which was associated with as little as 1° improvement in the Cobb angle over a 6-month treatment period. The MID based on changes in LC in participants reporting a GRC ≥2 (who rated their change as “a little bit better” to “a great deal better”) was an improvement of 1.3°. The ROC curve analysis revealed a similar MID estimate, a 1° improvement in the LC, by selecting the cutoff best predicting patients perceiving an improvement. Both of these estimates are smaller than the SEM of manual Cobb measurements (5°) or computer-based measurements (2.5°) [24].

The Schroth approach primarily aims to improve the postural awareness to correct the misalignments imposed by the scoliosis. Schroth uses specific corrective breathing, auto-correction [11] consisting of self-elongation and postural corrections specific for each curve pattern which is eventually integrated in daily activities. Much of the Schroth corrections target aspects other than the coronal plane deformity. Bracing and observation do not solely focus on improving the Cobb angle. Bracing aims to stop progression and observation reassures patient that they do not need more aggressive treatment yet. Therefore, the participants’ perceived positive change could arise in response to other effects of these conservative interventions regardless of the improvement in the Cobb angle. Research suggests that Schroth exercises improve posture in all three planes as measured by surface topography [16]. With the conservative interventions, the participant’s postural awareness and the overall body and trunk balance might have improved after the intervention and, some participants might have perceived improvement unrelated to the changes in the Cobb angle.

Some participants with AIS experience back pain and discomfort [34–37], as well as decreased function [35, 36], self-esteem, [38] and mental health concerns [39, 40]. The severity of these changes is only weakly related to the Cobb angle especially in curves severities treated conservatively [8, 41]. It may be that these outcomes, which are not routinely monitored in scoliosis research are being positively affected under the influence of the Schroth therapy [16, 42]. Other impairments, signs and symptoms of scoliosis could be affected by the conservative intervention, which in turn may be determinants of the perceived clinically important changes. This topic could be the focus of future research.

Although SOSORT suggests esthetics, quality of life, disability, and back pain as the most important outcomes to monitor in the treatment of patients with scoliosis, the Cobb angle remains the most widely used and accepted outcome in scoliosis care [43]. Tracking Cobb angle change makes most sense in surgery, where the primary goal is curve correction/stopping curve progression [44]. In contrast, the main objective of conservative treatments, including bracing and PSSE, is preventing progression in order to avoid further deterioration of signs and symptoms of scoliosis as well as directly addressing those signs and symptoms, with an ultimate goal to prevent surgery [4, 43]. In conservative care it is therefore logical to supplement the Cobb disease process outcomes with patients centered outcomes [45]. Patients undergoing conservative treatments constitute different populations with different specific characteristics and needs [46]. Patients under observation and surgical candidates could be perceived as two extremes along a continuum of treatment, where patients under observation may present with little to no concerns about scoliosis depending on individuals. Patients undergoing surgery are on the other side of the spectrum presenting with severe consequences due to scoliosis. In-between are the patients treated conservatively with obvious signs and symptoms of scoliosis but not as severe as to require surgery [47]. Outcomes deemed clinically important may vary along the continuum of treatment.

The current study was designed to determine whether the patients with Schroth PSSE added to the standard of care and those receiving standard care alone perceive important change beyond the simple two-dimensional Cobb angle change. Twelve participants in the Schroth and none in the Control group were classified as truly improved, while 15 participants in the Control and one in the Schroth group were classified as truly deteriorated. In our study, 17 patients in each group wore a brace which shows that the randomization was successful. Given above mentioned, and the fact that the brace did not have a significant main effect, as shown previously, [12] we concluded that the effect on the outcome was from the added treatment, i.e. Schroth PSSE.

Our results suggest that adolescents with AIS in our study who were undergoing conservative treatment, experience a positive change in the state of their backs even if the Cobb angle has not improved beyond the conventionally accepted threshold of 5° or the 2.5° SEM of our Cobb measurement approach [2]. Future studies are needed to determine if this observation is generalizable. The purpose of using a Cobb angle in this study was to show that while it is generally reliable and most commonly used to assess the effectiveness of a treatment, it does not appear to be what drives the patient perception of their back improvement. Patients often perceive changes in their bodies, their self-image and their sense of control related to their medical condition that is not reflected in the most commonly used clinical indicators such as Cobb angles. This paper supports this by finding that patients report improvement despite little or no change in their radiographs. Therefore, our understanding of what participants consider important when undergoing a conservative management of scoliosis should be improved.

## Limitations

Key limitations of this study are the relatively small sample size, intermediate follow-up duration and there is still a need for a validation of the MID estimates. Only 40 of 50 enrolled patients were analyzed because six dropped out and did not provide a GRC and four additional radiographs were missing. One control relocated and another control travelled for 3 months. Four Schroth participants dropped out because of time constraints. The radiographs were missing because the treating clinician did not order them, or they were acquired too late. The reasons for missing were unrelated to outcomes. Therefore, we believe that the sample was representative of the full dataset.

Generally, 6-month follow-up is considered a short follow-up in intervention trials [48]. However, the Cochrane Back and Neck review group distinguishes the following follow-up terms in exercise trials: short-term (closest to 4 weeks), intermediate (between 4 weeks and 1 year) and long-term (closest to 1 year). For surgery trials relating to back and neck, however, they suggest 2- or 5-year follow-up. [49]. This suggests that exercise and surgery trials for scoliosis should interpret the outcome timepoints differently, because the purpose and nature of these two interventions is clearly different. Patients with AIS are typically monitored routinely until 1–2 years after reaching skeletal maturity [48]. What is perceived as clinically important by patients over this intermediate interval may be different than what is considered important over the full follow-up duration until maturity. Nevertheless, we argue that this 6-month duration is important for adolescents who are either attending regular exercise sessions, wearing a rigid brace full time or waiting for the next exam while under observation wondering if their condition progressed. Future research may be needed to demonstrate whether perceiving important short and intermediate terms improvements has long term implications on motivation and compliance with treatments and final outcomes.

There still is controversy concerning what is the best methods to estimate the MID. It is therefore, recommended to use different methods and triangulate results to arrive at a MID range or weighted estimate based on multiple methods. It is also recommended to obtain validation of estimation provided by original estimation attempts and to verify whether estimates in one context can generalize to others (anchor choice, different conservative treatments, follow-up duration, sample characteristics). Estimates presented in the present study should be validated in future research. Further, patients receiving a brace and under observation at our institution are usually told that stabilization not improvement is the goal of the care they receive. Therefore, it is possible that some patients in the study perceived positive outcome event when the Cobb angles showed little to no change. Future studies could examine effect of using different GRC cut-offs to estimate the MID.

This was a secondary analysis of a randomized controlled trial that investigated the effect of the Schroth exercises added to standard of care on curve severity, muscle endurance and quality of life as compared to standard of care alone consisting of observation or bracing. In the RCT, ethically, we could not withhold bracing if indicated for a patient. The present study did not focus on the effectiveness of the conservative treatments compared, only on determining MID using the combined sample. Our RCT study was not powered to allow subgroup analysis of the effect of different conservative treatments. The continuing multicenter SETS trial should allow for these comparisons (ClinicalTrials.gov, NCT01610908).

## Conclusion

Participants undergoing Schroth treatment added to the standard of care or standard care alone over 6 months perceived a positive change in the state of their backs even if the Cobb angle did not improve beyond the accepted threshold of 5°. The perceived improvement in the overall back status appears to be due to improvements in outcomes other than the two-dimensional Cobb angle. This study warrants investigating further outcomes that might be more relevant in determining what the patients with AIS perceive as important to monitor during the conservative treatment.

## Data Availability

A spreadsheet with data that support the findings of this study is available in PLOS One as supplementary material to a related article with the identifier “S2 File. Excel file containing study data. Doi:10.1371/journal.pone.0168746.s003

## Abbreviations

AIS: Adolescent idiopathic scoliosis
AUC: Area under curve
GRC: Global Rating of Change
LC: Largest curve
MID: Minimal important difference
PSSE: Physiotherapeutic Scoliosis-Specific Exercises
RCT: Randomized controlled trial
ROC curve: Receiver operating characteristics curve
SEM: Standard error of measurement
SOSORT: Society on scoliosis orthopaedic and rehabilitation treatment
SRS: Scoliosis Research Society
